# Relative costs and effectiveness of treating uncomplicated malaria in two rural districts in Zambia: implications for nationwide scale-up of home-based management

**DOI:** 10.1186/1475-2875-10-159

**Published:** 2011-06-09

**Authors:** Pascalina Chanda, Busiku Hamainza, Hawela B Moonga, Victor Chalwe, Patrick Banda, Franco Pagnoni

**Affiliations:** 1 Department of Public Health and Research, Ministry of Health Headquarters, Lusaka, Zambia; 2 Operational Research Unit, National Malaria Control Centre, Lusaka, Zambia; 3 Parasitology Unit, National Malaria Control Centre, Lusaka, Zambia; 4 Department of Clinical Sciences, Tropical Diseases Research Centre, Ndola, Zambia; 5 Department of Planning and Policy, Ministry of Health, Lusaka, Zambia; 6 UNICEF/UNDP/World Bank/WHO Special Programme for Research and Training in Tropical Diseases, World Health Organization, Geneva, Switzerland

## Abstract

**Background:**

Malaria case management is one of the key strategies to control malaria. Various studies have demonstrated the feasibility of home management of malaria (HMM). However, data on the costs and effectiveness of artemisinin-based combination therapy (ACT) and rapid diagnostic tests via HMM is limited.

**Method:**

Cost-effectiveness of home management versus health facility-based management of uncomplicated malaria in two rural districts in Zambia was analysed from a providers' perspective. The sample included 16 community health workers (CHWs) and 15 health facilities. The outcome measure was the cost per case appropriately diagnosed and treated. Costs of scaling-up HMM nationwide were estimated based on the CHW utilisation rates observed in the study.

**Results:**

HMM was more cost effective than facility-based management of uncomplicated malaria. The cost per case correctly diagnosed and treated was USD 4.22 for HMM and USD 6.12 for facility level. Utilization and adherence to diagnostic and treatment guidelines was higher in HMM than at a health facility.

**Conclusion:**

HMM using ACT and RDTs was more efficient at appropriately diagnosing and treating malaria than the health facility level. Scaling up this intervention requires significant investments.

## Background

Malaria case management is one of the key strategies to control malaria [1]. The adoption of new effective artemisinin-based combination therapy (ACT) in Africa has began to show positive health impacts in terms of malaria morbidity and mortality reduction [1,2] with some countries contemplating malaria elimination [1]. Additionally, the introduction of rapid diagnostic tests (RDTs) for malaria has changed the approach to malaria diagnosis. Presumptive treatment of fevers as malaria is no longer encouraged because it leads to misdiagnosis of the disease and irrational drug use [3]. With the adoption of RDTs in routine health services, malaria confirmation is no longer a preserve of areas with laboratory services. RDTs are increasingly gaining attention for their practical use given the limitations of scaling up microscopy services [4]. RDT diagnosis of malaria has been said to be rapid and requiring less inputs than microscopy and no specialized personnel is required to perform the tests [5,6]. This has potentially improved patient access to diagnosis services, especially in peripheral health centres [6].

In spite of all these achievements, access to prompt and effective case management still remains a challenge. It is for this reason that WHO recommended the adoption of the home management of malaria (HMM). HMM has been shown to be feasible and acceptable [7]. Additionally various approaches for scaling up HMM include pharmacists, villages volunteers or shopkeepers [8]. The choice of strategy depends on the health system context.

Malaria in Zambia is a major cause of visitation and hospitalization. In 2009, approximately three million cases and 2,000 deaths were recorded from health facilities countrywide [9]. ACT and RDTs in Zambia are provided free of charge in the public sector both in rural and urban areas up to health facility level. In urban areas, some private health facilities do provide malaria and other health services to a less extent. However, in rural areas, the people mostly depend solely on the available public health facilities. Sometimes, the facilities are in distant locations, which create geographical access barriers. In areas where the health facilities exist, the lack of adequate human resources sometimes leads to delays and long queues.

Community health workers (CHWs) form part of the health system in the rural areas. Their main mandate, until recently, was in health education. However, due to the growing demand for patient care, plans are underway to scale up HMM with ACT and RDTs using CHWs. CHWs are able to interpret instructions correctly on how to use RDTs [10] and they can safely prescribe ACT.

A study was carried to evaluate the cost and effectiveness of managing uncomplicated malaria with ACT and RDTs using CHWs in comparison with the health facility model.

## Methods

### Study design

This was a cost-effectiveness evaluation of the management of uncomplicated malaria using the HMM strategy *versus *health facility-based management following the standard malaria treatment guidelines for Zambia. The provider perspective was used because malaria services are offered free of charge to the user since malaria is part of the basic health care package in Zambia. The basic health care package is a package of basic health services for common diseases, which are provided free of charge in government (public) owned health facilities. The government is the main health provider in the country. The main outcome measure was the proportion of uncomplicated malaria cases correctly diagnosed and treated according to national malaria case management guidelines.

### Study population and duration

The study included all age groups who sought care through the CHWs and the health facility in the study sites between January and December 2009. The study period covered both high and low transmission seasons. Both strategies involved use of diagnostic testing and treatment with ACT. In Chongwe district, nine CHWs with a total catchment area of 16,079 were included in the study, while in Kalomo seven CHWs surrounded by 18,279 people were included in the study. The CHWs were selected based on prior IMCI training and involvement in malaria at the time of the study. In both districts, the villages served by CHWs are far from the health facilities but the patients have a choice whether to use the CHWs or the health facility as the first point of care. The health facilities included in the study were those in the vicinity of the selected CHWs in each district; eight in Chongwe and seven in Kalomo districts repsectively.

### Study sites

The study was conducted in Chongwe and Kalomo district as part of a main study to evaluate the feasibility and effectiveness of the management of uncomplicated malaria using ACT and RDTs in the home management of malaria via CHWs. Chongwe district is located in Lusaka Province, an area which is typically rural and experiences moderate malaria transmission. The district is estimated to have a population of about 157,664 inhabitants, with 26 health facilities and an annual malaria incidence estimated at 130/1,000 population in 2008 [9]. Kalomo district on the other hand is situated in the southern Province, with a population of about 181,379 inhabitants. The district has 24 health facilities and the annual malaria incidence was estimated to at 82/1,000 population [9]. Both districts are implementing the user fees removal policy (thus patients are not expected to pay any fees for the basic health care package).

### Description of the interventions under comparison

#### Home management of malaria delivered by CHWs

This strategy involved training the already existing CHWs on the use of RDTs and ACT in the management of uncomplicated malaria. Further training was provided on the management of biological wastes and sharp objects, such as lancets. Additional orientation on drug and logistics management was given. The CHWs were then provided with malaria registers and medical supplies and re-deployed to their areas of operation. Any fever patient who reported to the CHWs was supposed to be subjected to a finger prick to ascertain malaria status. Complicated malaria cases and non-malaria febrile cases were referred to the nearest health facility for further management. All services were provided free of charge, in line with the country policy on health care financing. Pre-packaged AL was the ACT of choice and HRP-II RDTs were used according to the country stocks. The management of the stocks of AL, RDTs and other consumables was the responsibility of the CHWs under the supervision of the health facility as per routine operation guidelines. Replenishment of drugs and logistics were made by the health facility once the CHW made a request using a specially designed logistics form for RDTs and ACT. Each CHW was provided a new bicycle for transport (this is the standard form of transport for CHWs in Zambia). The study team conducted monthly supervisions to ensure data completeness of the malaria registers.

#### Health facility management of malaria delivered by professional health workers

Under this strategy (standard practice), a clinical officer, environmental health technician or nurse is in charge of managing health facilities at any of the selected 15 health facilities. The health facilities were selected on the basis of being in the locality of the CHWs included in the HMM strategy described above. These health workers were prior to the study already trained in malaria case management using the new guidelines (confirmation plus use of ACT). At the health facility two options are available for diagnosis: microscopy or RDTs. The patient flow has already been described elsewhere [11]. The health workers received additional orientation (refresher) on new treatment guidelines, supervision of CHWs and logistics management. Pre-packaged AL was the ACT of choice and HRP-II RDTs were used according to the country stocks. Other anti-malarials at health facility level included SP for IPT and quinine for severe malaria. Chloroquine is no longer part of the anti-malarial list for health facilities in Zambia. The health facility level manages both complicated and uncomplicated malaria using a tiered referral system. Additional referrals from CHWs were also managed accordingly. Malaria diagnosis and treatment data was recorded in the out-patient department (OPD) malaria register. The study team carried out monthly supervision to ensure data completeness for the purposes of the study.

### Data collection

Malaria registers were used by both the health facility personnel and CHWs. All the patients who were managed during the study period were recorded in these registers. The method of diagnosis and type of treatment was also recorded. Secondary data from published literature and official Ministry of Health records were used for costing information as shown in Table 1.

**Table 1:** Parameter Assumptions and Data Sources

Description	Assumption	Source
**Exchange Rate** **(annual average for 2009)**	1USD = ZMK4900	http://www.aonda.com

**Discount rate**	5%	MOH Planning Unit

**Overhead costs**	15% (Of district recurrent expenditure)	District Health Office (DHO)

**Personnel costs**	Gross earnings (Taken from central level)	MOH/DHO

**Cost of drug**	AL = 1.00 USD SP = 0.18 USD Quinine = 0.84 USD	NMCC, (weighted average cost per person/course including storage and distribution costs).

**Cost/test**	RDT = 0.6USD Microscopy = 1.00 USD	NMCC (excludes personnel and capital costs).

**Sensitivity (RDTs)**	95.4%	[19,20].

**Prevalence (national)**	27.0%	[13]

**Annual average malaria prevalence at OPD**	Kalomo = 26% Chongwe = 22%	[9,21]


The ingredient approach combined with step-down approach to costing was used to estimate average costs per year in line with methods for costing health programmes [12]. Step-down costing was used in personnel, administrative and some capital cost centres. Inventories on capital and recurrent costs related to malaria diagnosis and treatment at facility and community level were made. Procurement reports, receipts, action plans and market prices were used to measure and value the resources used.

Capital resources (i.e. items which have a useful life of more than one year) were annualized based on the replacement value, its estimated useful life and the official discount rate used in Zambia (5%). Capital costs at health facility level included equipment, vehicles and buildings. The allocation of capital costs to malaria diagnosis was performed by estimating an allocation factor per facility based on malaria OPD utilization. Other facility level costs have already been described [11].

### Recurrent costs

Personnel costs were measured based on number and categories of each type of staff (nurse, clinical officer, medical doctor, community health worker, etc) and their respective annual salaries. These were then allocated based on the utilization of facilities by suspected malaria patients. Shared recurrent costs for supplies and utilities were valued and allocated based on the facility utilization factor. However, costs unique to malaria (such as cost of the diagnostic technique) were fully allocated as such. The parameters and assumptions used in the analysis of costs and outcomes are summarized in the Table 1.

#### Outcome measure

The outcome measure was the proportion of cases appropriately diagnosed and treated. Appropriate diagnosis was malaria confirmation by either RDT or microscopy. Appropriate treatment included: AL first line treatment for uncomplicated malaria for all age groups except for children weighing 5 kg or less in whom SP is prescribed; Quinine for complicated malaria and no anti-malarials in cases with a negative test outcome.

### Average cost effectiveness and incremental cost effectiveness analysis

Malaria related costs were identified and analysed. Cost profiles included, drug costs, RDT costs, other consumables, transport, personnel time and other relevant inputs for both arms. The total cost of diagnosis was estimated using the number of cases tested by RDT or microscopy multiplied by the unit cost of diagnosis for each strategy. The average cost per case tested was determined by dividing the total costs of diagnosis by number of cases tested by RDT and microscopy. The average cost per case treated was determined by dividing the total costs of treatments by number of cases treated under each strategy.

#### Cost effectiveness analysis

The main outcome measure was the number of malaria cases appropriately diagnosed and treated according to national case management guidelines..

Thus cost-effectiveness = Total cost/number of malaria cases appropriately diagnosed and treated under each alternative.

#### Incremental cost-effectiveness

The incremental cost per additional case appropriately diagnosed and treated was calculated based on the changes in the costs and effects of moving from the strategy that costs less per patient diagnosed and treated to the next alternative.

Thus: ICER = change in cost/change in cases appropriately diagnosed and treated.

#### Ethical consideration

Ethical approval was obtained from the Tropical Disease Research Ethics Committee. Further clearance was obtained from the Permanent Secretary of the Ministry of Health.

## Results

The average utilization of CHWs by the target population in the two districts was estimated at 29% (35% in Chongwe and 23% in Kalomo district). The utilization rate was estimated using the catchment population of the CHWs in each district as the denominator and the number of visits to the CHW as the numerator. The catchment population under consideration was 16,079 in Chongwe and 18,279 in Kalomo. The crude parasite prevalence was 24% and 26% among CHW and health facility visits respectively. These figures are within the national estimates of 27% from the national population based parasitological surveys [13].

At community level no microscopy testing was done, all the cases were diagnosed using RDT while at health facility level either microscopy or RDT was used. This is in line with national policy of introducing only RDTs at community level but health facilities can use whichever test is available at the time of a patient visit. The proportion of patients in whom a confirmatory test was performed was higher at community (97%) than at health facility level (56%) as shown in Table 2.

**Table 2:** Average cost per case appropriately diagnosed

Variable	HMM	Health facility
**Number of cases**	9,847	53450

**Number tested with RDT**	9,552.	24587

**Number tested with microscopy**	0	5345

**Number tested clinically**	295.00	23518

**Proportion of fever cases in whom test is used**	97%	56.00%

**Total tested with either RDT or microscopy**	9,552.00	29932

**Total cost of diagnostic test (USD)**	38,112.48	159387.90

**Proportion of cases appropriately tested**	**97%**	**56%**

**Average cost per case appropriately tested (USD)**	**3.99**	**5.33**


The average cost per case appropriately tested was higher at health facility than at community level. This is because personnel and capital costs are higher at health facility level. The current HMM strategy uses CHWs who have been working on voluntary basis and thus are not salaried. However, the costs of RDTs and ACT are the same at HF and HMM level because they use the same national procurement and distribution system.

### Treatment

More negative cases were treated with anti-malarial at health facility than at community level. The proportion of cases correctly treated was higher at community than at health facility level as shown in Table 3. 30% negative on test were treated with anti-malarial at health facility. Among the cases testing positive at facility level 52% were treated with AL, 33% with SP and 10% with quinine. Additionally, 5% of the cases testing positive at health facility level were not prescribed an anti-malarial.

**Table 3:** Cost per case appropriately diagnosed and treated

Variable	HMM	Health facility
**Number positive for malaria**	2,318.00	7782

**Parasite prevalence**	24%	26%

**Number treated with AL**	2,282.00	4047

**Number treated with SP**	16.00	2568

**Number referred for quinine**	20.00	778

**Negative for malaria treated with anti-malarial**	13.00	6645

**Negative for malaria not treated with anti-malarial**	7,516.00	15505

**Proportion of cases appropriately treated**	**100%**	**43%**

**Total cost of anti-malarial treatment (USD)**	2,303.22	29,845.76

**Total number of cases correctly treated**	9,834.00	23,287

**Cost per case appropriately treated (USD)**	**0.23**	**1.28**

**Cost per case appropriately diagnosed and treated (USD)**	**4.22**	**6.61**

**Incremental effectiveness (HF to HMM)**	**0.57**	

**Incremental costs (HF to HMM**	**2.38**	

**ICER**	**4.18**	


The incremental cost-effectiveness ratio was USD 4.18 per case appropriately diagnosed and treated. This is the cost required to increase the number of cases appropriately diagnosed and treated (that is to introduce HMM with ACT and RDTs at community level in the rural areas where it is applicable).

### Estimation of costs of scaling up HMM with ACT and RDTs in rural parts of Zambia

The assumptions for population were based on the 2010 census figures for Zambia (CSO Monthly Bulletin 2011). The census figures indicates that of the 13,046,508 persons in Zambia, about 7,978,274 (61%) were in rural areas. According to this study, it was estimated that 35% and 23% of catchment population utilized the CHWs for malaria in Chongwe and Kalomo respectively. These figures were used to estimate the number of CHWs required to scale up HMM to meet the target population in the rural areas. The estimates were made for a ratio of 1CHW per 500 persons in the rural areas. In the scenario where the average 29% of the rural population utilized the CHWs, the scale-up costs were estimated to be about USD 9,763,811.72. This represents about a quarter of the annual national malaria budget.

Scenarios were also made for made for 20% and 35% utilization as shown in Tables 4 and 5. The costs of scaling up were USD 6.7 million for 20% and USD11.8 million for 35% utilization. These costs may be reduced by increasing the CHW to patient ratio from 1:500 to 1:1,000.

**Table 4:** Estimated costs of scaling up HMM assuming 29% of the rural communities utilize CHWs

1CHW:500	
**Rural Population for Zambia (CSO 2011)**	7,978,274.00

**Expected @ 29% (average of 35%&23%) observed in study**	2,313,699.46

**Cost per case appropriately diagnosed and treated**	4.22

**Scale-up costs (USD)**	9,763,811.72


**Table 5:** Estimated costs of scaling up HMM assuming 20% and 35% of the rural communities utilize CHWs

1CHW:500	
**Population Rural for Zambia (CSO 2011)**	7,978,274.00

**Expected utilization @ 20%**	1,595,654.80

**Cost per case appropriately diagnosed and treated**	4.22

**Scale-up costs (USD)**	**6,733,663.26**

**Expected utilization @ 35%**	2,792,395.90

**Cost per case appropriately diagnosed and treated**	4.22

**Scale-up costs (USD)**	**11,783,910.70**


## Discussion

The community approach could provide an effective and cost-effective way of increasing access to prompt and effective case management. HMM with RDTs and ACTs can be scaled up even in resource constraint settings, such as Zambia where there is also a looming human resource crisis. It also provides more evidence on the economical and health benefits of introducing ACT and RDTs in the HMM strategy.

It is important to point out that better practices were reported in the HMM model than at health facility level. CHWs adhered to case management guidelines and this consequently resulted in more cases appropriately diagnosed and treated than at facility level. The findings also bring out an improtant aspect that the availability of commodities for malaria control should not be the only bottleneck to be improved upon in an effort to deliver effective case management. Traning and mentoring is key to ensure that heath workers adhere to good case management so that they utilize the available tools correctly. Otherwise the cost-effectiveness of ACT and RDTs are negated by the poor practice of health workers [14]. Non adherence to malaria diagnostic test result has already been documented to be a problem among health workers in Zambia [15,16]. The training model for CHWs used in this study can also be scaled up to large scale health facility level so as to introduce good practices at health facility level. This has the potential to improve not only malaria case management but overall patient management as well. This model of training was used in a health facility study in one district and similar good case management practices were attained [2]. This is because not prescribing anti-malarials to non-malaria fevers, provides an opportunity for patients to be invstiagated for another cause of illness. HMM with ACT and RDTs is thus not only economical but it can potentially lead to better health outcomes. In Kenya, a study demonstrated that shopkeeper trainings improved the proportion of cases taking appropriate anti-malarial doses [8].

The cost per case appropriately diagnosed and treated through HMM is lower than cost which have been reported by other studies for scaling up HMM using various strategies. In Ghana, IPTc via community based voulunteers found the least cost effective method to be about USD 8.19. HMM without diagnosis has been demonstrated not to have better outcomes than the facility level [17]. This seems to emphasize the need to adopt the strategy of including the diagnosis component in HMM as has been recommended by other researchers [8]. RDTs are attractive and easy to scale up at community level than microscopy because less capital invetsments are required [5,6]. Furthermore, in Zambia, it has been demonstarted that RDTs are cost effective in the management of malaria at facility level [11]. The negative effects of false negatives are minimized by use of HRP-II based RDTs which have been shown to overestimate the burden of malaria by 10% [18].

The scale-up costs found in this study are substantial at the beginning of the programme, but the maintenance costs are feasible and can be managed by district level management. This is because in the case of Zambia, both ACT and RDTs are centrally procured and thus the HMM strategy does not require its own logistics and management system. The CHWs are also already part of the health care system and do receive baseline training in health programmes. This level of community involvement provides a vehicle for the delivery of HMM by adding effective tools such as RDTs and ACT instead of treating malaria patients with SP based on fever. Thus other malaria programmes contemplating HMM should consider the health system context and review existing channels which can be used to have an effective HMM strategy.

The cost per patient appropriately diagnosed could change upwards if CHWs were to be salaried, however, the level of increase would be determined by the volume of non-malaria services that CHWs may be allowed to offer if salaried. Additionally, capital investments such as screening rooms or booths for CHWs to work in may also positively change the unit costs found in the HMM strategy. However, none of these would render the HMM strategy to be less effective.

Further areas for research include the impact of HMM strategy on the outcomes of non-malaria fevers and the effectiveness of the training model proposed in this study in a multicentre study.

## Conclusion

HMM was more cost-effective for management of uncomplicated malaria in rural areas than the standard of care at health facility level (USD4.22 versus USD6.61). Utilization and adherence to both diagnostic and treatment guidelines was higher in HMM strategy than at health facility level. Scale-up costs for HMM would depend on the expected utilisation and the desired patient to provider ratio.

## Competing interests

The authors declare that they have no competing interests.

## Authors' contributions

PC was responsible for the proposal development, study administration, data analysis and manuscript development. BH, MH and VC participated equally in proposal development, data collection, data entry and analysis and manuscript development. PB participated in data collection, monitoring and reviewed the manuscript. FP provided invaluable input at proposal development, data analysis and manuscript development. All authors read and approved the final manuscript.
